# 

**Published:** 1892-06

**Authors:** 


### TABLE OF CONTENTS.

### ORIGINAL COMMUNICATIONS.

PAGE

Shock and Collapse resulting from Dental Operations. By JOHN S. MARSHALL, M.D., Chicago, Ill. . . . . . . . . . . . . . . . . . . . . . . 405
Description of a Case of Regulating. By DR. H. F. HAMILTON, Boston . 416
A New Illuminating Method by the Use of Zirconite. By DR. W. R. PATTON, Cologne, Germany . . . . . . . . . . . . . . . . . . . . . . . 418
The Dentist's Hygiene. By E. DE TREY, D.D.S., Basel, Switzerland . . 422
Nitrite of Amyl. By HENRY A. KELLEY, D.M.D. . . . . . . . . . . . . 430
The Physiognomical Significance of the Teeth. By GEORGE W. WARREN, D.D.S., Philadelphia . . . . . . . . . . . . . . . . . . . . . . . 437

### REPORTS OF SOCIETY MEETINGS.

New York Odontological Society . . . . . . . . . . . . . . . . . . . 439
American Dental Association . . . . . . . . . . . . . . . . . . . . 449
American Academy of Dental Science . . . . . . . . . . . . . . . . 459

### EDITORIAL.

Professional Jealousy . . . . . . . . . . . . . . . . . . . . . . . 470
Dr. C. M. Richmond's Papers on Crown- and Bridge-Work . . . . . . . 473
The World's Columbian Dental Congress . . . . . . . . . . . . . . . 473
To our Contributors . . . . . . . . . . . . . . . . . . . . . . . . 474
Correction . . . . . . . . . . . . . . . . . . . . . . . . . . . . 474

### OBITUARY.

Dr. Charles A. Kingsbury . . . . . . . . . . . . . . . . . . . . . 474

### CURRENT NEWS.

The World's Columbian Dental Congress . . . . . . . . . . . . . . . 475
Pennsylvania and New Jersey State Dental Societies' Joint Union Meeting 483
American Dental Society of Europe . . . . . . . . . . . . . . . . . 484
The Missouri State Dental Association . . . . . . . . . . . . . . . 484
Massachusetts Dental Society . . . . . . . . . . . . . . . . . . . . 484

